# The influence of real-time quantitative feedback and verbal encouragement on adults’ performance in maximal and explosive strength and power in bench press exercise

**DOI:** 10.3389/fphys.2024.1329432

**Published:** 2024-08-20

**Authors:** Martin Pacholek

**Affiliations:** Department of General Studies, Prince Sultan University, Riyadh, Saudi Arabia

**Keywords:** motivation, resistance training, students, quality education, stimuli

## Abstract

**Background:**

In sports practice, a wide array of verbal and non-verbal stimuli can elicit diverse motivations and performance changes. Therefore, the primary objective of this study was to compare the impact of various stimuli on maximal strength and power in bench press exercises.

**Methods:**

This study involved 48 university students (average age 20.5 ± 2.8 years; body mass 80.1 ± 20 kg; height 174.6 ± 6.7 cm; BMI 26.2 ± 6 kg/m2) who engaged in an 8-week resistance training program. The students were randomly divided into three experimental groups and one control group. The first group received real-time quantitative feedback (RF) on their power output during the bench press exercise, the second group received verbal encouragement (VE) from an instructor, and the third group exercised without any external stimulus (WS). The control group (CG) underwent only pre- and post-measurements. To compare differences in strength parameters among groups a Two-Way Repeated Measures ANOVA was applied.

**Results:**

The results revealed significant improvements in the mean weight for one repetition maximum in the real-time quantitative feedback group (5 kg, 9.76%, *p* = 0.001, d = 0.529) and the verbal encouragement group (5.42 kg, 11.51%, *p* = 0.001, d = 1.201). Positive changes were also observed in the mean power at 20 and 30 kg for the RF, VE, and WS groups, but at 40 kg, significant improvement was only seen in the real-time quantitative feedback group (247 W, 31.30%, *p* = 0.001, d = 1.199).

**Conclusion:**

These findings underscore the effectiveness of selected stimuli in enhancing maximum strength and power during bench press exercises, with real-time quantitative feedback proving to be the most effective stimulus for improving both maximal strength and power.

## 1 Introduction

Body movements are patterns of responses to recognized stimuli (Melzer et al., 2019). These stimuli, which can be visual, kinesthetic, auditory, or a combination of multiple senses, are perceived by individuals (Morgan, 2019). Learning occurs by conditioning responses to specific stimuli (Bam, 2016). Applied behavior analysis has increasingly emphasized the significance of stimuli in regulating human behavior (Winnick and Porretta, 2017).

In the context of physical activity settings, a growing body of research has provided evidence for the influential role of positive feedback as a stimulus in shaping perceptions of competence and intrinsic motivation (Schunk, 1995; Reinboth et al., 2004; Nicaise et al., 2006). The feedback provides information about the objective or subjective results, or the quality and quantity of movements during or after the performance (Kangalgil and Özgü, 2018). According to Doig (2001), feedback is a helpful tool for individuals to achieve desired outcomes according to certain criteria. Badami et al. (2011) have differentiated between two types of feedback employed in teaching and coaching: intrinsic feedback (feedback that arises from learners’ sensory systems during and as a consequence of their performance) and augmented feedback (feedback received from an external source that supplements the learner’s sensory information). The utilization of these two types of feedback in conjunction assists students and athletes in thriving and enhancing their performance (Smither et al., 2005).

The feedback provided by teachers and coaches significantly influences the achievement of students and athletes in physical education and sports settings (Mouratidis et al., 2008). Practitioners utilize feedback to teach correct movements and skills, enabling students and athletes to assess their performance (Lund and Kirk, 2019). Feedback serves as a tool to rectify mistakes and impact an individual’s motivation levels (Coker, 2013). It can be delivered in various forms, including verbal, visual, or written, and does not always require intricate details to enhance motivation (Strube and Strand, 2015).

Koka and Hein (2003) discovered that perceived feedback played a pivotal role in students’ perceptions of competence and intrinsic motivation. Similarly, the sports literature is abundant with evidence highlighting the crucial role of positive feedback from coaches in athletes’ perceptions of competence and intrinsic motivation (Amorose and Horn, 2000; Chelladurai and Saleh, 1980). One of the most straightforward methods for enhancing motivation and competitiveness is through the effective use of feedback (Wilson et al., 2017; Weakley et al., 2020).

Real-time quantitative feedback is a powerful tool for creating a competitive environment that can lead to acute improvements in performance and, over time, drive adaptation (Argus et al., 2011; Randell et al., 2011; Wilson et al., 2017). This straightforward method is often overlooked but has demonstrated a significant impact on the development of strength and power (Weakley et al., 2020). Studies (Argus et al., 2011; Randell et al., 2011; Singh, 2016; Wilson et al., 2017) have shown that real-time quantitative feedback results in higher movement velocities and can enhance training performance by approximately 3%–6%. It enables individuals to train closer to their optimal capacity (Figoni and Morris, 1984; Graves and James, 1990; Kellis and Baltzopoulos, 1996; Owen, 2014).

Another method for motivating individuals is through verbal encouragement (VE). A recent study has shown that receiving verbal encouragement in conjunction with the presence of practitioners during exercise can lead to enhanced performance (Keegan, et al., 2010). It is also recognized that implementing verbal or visual feedback can yield the same positive effect on performance as having a coach present (Weakley, 2020). In the same study, it is mentioned that verbal encouragement yields comparable improvements in strength training when compared to receiving kinematic feedback. This underscores the importance of this stimulus in maintaining motivation during practice (Standage et al., 2003). However, it is worth noting that a study by Campenella et al. (2000) found no significant effect of verbal encouragement on strength efforts. This indicates that there is limited evidence regarding the effectiveness of verbal encouragement in strength training, especially considering that most studies have focused on endurance performance (Bickers, 1993; Moffatt et al., 1994; Andreacci et al., 2002; Neto et al., 2015). Furthermore, several exercise testing guidelines have included specific steps for using verbal encouragement, but without solid theoretical or empirical justification. Additionally, there has been limited research defining effective verbal encouragement in terms of content, tone, loudness, timing, and frequency of delivery (Midgley et al., 2018).

Until now just a few studies have investigated the chronic effect of resistance training with different stimuli or feedback (Weakley et al., 2023). Therefore, the primary objective of this study is to compare the effectiveness of different stimuli, namely, real-time quantitative feedback, verbal encouragement, and no external incentives. This research aims to shed light on the impact of these stimuli on student motivation and performance, providing valuable insights for both scientists and practitioners. The study is unique in its approach, as it combines several elements: a comparative analysis of selected stimuli within the training program, a group of students, and customized training loads tailored to each participant.

The hypothesis posited that all experimental groups would exhibit significant improvements in maximal strength (one repetition maximum-1RM) and explosive strength (mean power) during bench press exercises, in contrast to the control group (CG). Furthermore, it was expected that the group utilizing real-time quantitative feedback would experience significantly greater improvements compared to the other groups following the implementation of the selected resistance program. Lastly, it was anticipated that the experimental groups would demonstrate greater enhancements in explosive strength as opposed to maximal strength upon completion of the selected resistance program.

## 2 Materials and methods

### 2.1 Participants

This study followed a blinded four-group–time-parallel experimental design, with the dependent variables being motoric abilities (maximal and explosive strength). The independent variables were perceived feedback during resistance training (real-time quantitative feedback, verbal encouragement). A total of forty-eight male students from Prince Sultan University participated in the study (average age 20.5 ± 2.8 years; body mass 80.1 ± 20 kg; height 174.6 ± 6.7 cm; BMI 26.2 ± 6 kg/m2), and all participants completed the training and measurements. They were all in good physical health, without any injuries, and refrained from taking supplements during the program. Every student had less than 2 years of experience with progressive resistance training and more than 1 month of experience with weightlifting in the gym. Therefore, the purposive sampling method was employed to select participants for our study. Students were informed about the testing procedures, training programs, and potential risks associated with these activities before the pre-test measurement of their explosive and maximal strength abilities. However, they were unaware of the specific interventions and were instructed not to engage in any exercise routines other than the selected program. Each student underwent pre- and post-measurements before and after an 8-week resistance program.

### 2.2 Instruments

Prior to the actual measurement, they had three practice attempts using a 20 kg barbell for the bench press test. The students were randomly divided into four groups, each one with 12 students. Three of these groups underwent resistance training programs in the gym, with different interventions or without any stimuli, while the fourth group (control group) underwent only pre- and post-measurements. The real-time quantitative feedback group received feedback from a device called FITROdyne Premium (FITRONIC, Bratislava, Slovak Republic) about their performance (power in the concentric phase) after each repetition. This feedback was both visible to the participant and verbally announced by the teacher or their peers. The verbal encouragement group received verbal encouragement from the teacher before each repetition and set. The verbal encouragement consisted of a single word, “hoop,” which was loudly spoken by the teacher before each repetition and concentric phase of the movements. The without any external stimulus group (WS) worked out without any feedback. All testing procedures, interventions, and training were administered by a single investigator. The experiment had a duration of 8 weeks, with sessions held twice a week for a total of 16 training units.

### 2.3 Procedures

**FIGURE 1:**
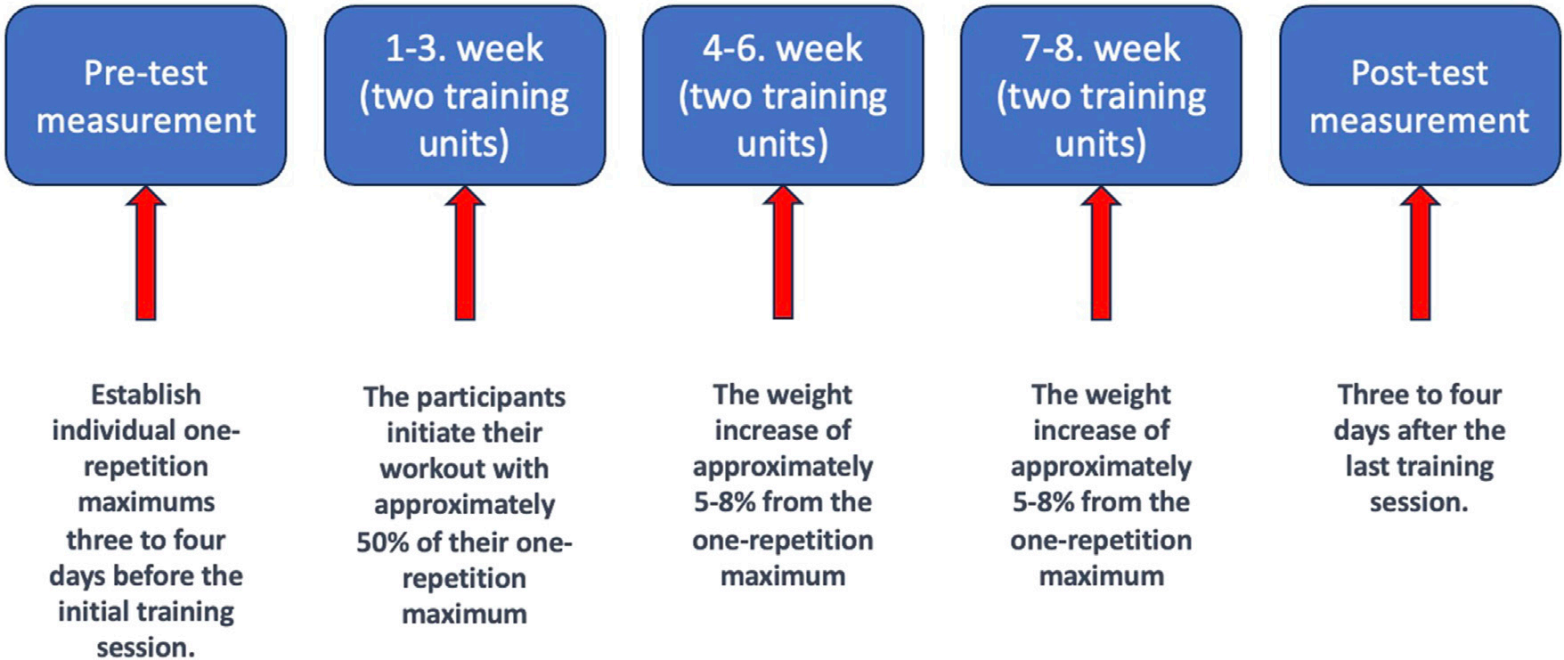
Training program.

Students received individualized training programs based on their pre-test measurements in the bench press (BP) exercise (Figure 1). Each participant began with a standardized warm-up, consistent for all participants, and then proceeded to work with weights starting at approximately 50% and concluding at around 65% of their one-repetition maximum (1RM). Every 3 weeks, weights were incrementally increased by about 5%–8% based on their individual programs. Interval rests between sets ranged from 3–6 min. The training program included two sets with 4-6 repetitions (based on their subjective feelings), emphasizing subjective maximal velocity during the concentric phase. Participants were instructed to stabilize the barbell on their chests for 3 s before beginning the next repetition of the bench press concentric phase. All participants had a minimum of 48 h of rest between training sessions.

The study adhered to ethical standards for human experimentation as outlined in the 1964 Helsinki Declaration and its subsequent amendments. Approval for the project was obtained from the Prince Sultan University Institutional Review Board (PSU IRB-2022-02-0104).

### 2.4 Data collection and processing

Students were instructed to abstain from engaging in physical activity for at least 2 days prior to the measurements and to avoid consuming solid meals within 2 h before testing. On the day of testing, participants followed a standardized warm-up routine, which included 1 min of running in place, dynamic stretching exercises for the upper body, and five sets of push-ups.

Following the warm-up, an assessment battery was conducted to assess maximal power and maximal strength. The students underwent strength testing to determine their one-repetition maximum (1RM) on the bench press (BP). The bench press is a commonly used exercise for developing upper body strength.

The investigator served as a spotter during the bench press measurements. Firstly, it was checked if participants had the correct grip on the bar (slightly wider than shoulder-width) and provided assistance with unracking the bar when necessary. Students then started the exercise with their arms fully extended and elbows locked. Then the bar was lowered until it touched the chest, followed by a concentric phase, lifting the bar back to full arm extension. A repetition was considered successful only if it included this full range of motion. Any deviation, such as failing to touch the chest or bouncing the bar, was regarded as an unsuccessful attempt. For safety reasons, the spotter remained positioned behind the bar throughout the exercise and intervened by assisting with lifting the bar off the participant if an attempt was unsuccessful or unsafe. However, during successful attempts, the spotter did not touch the bar (Wilk, et al., 2020).

Participants aimed to achieve maximum velocity during the concentric phase while lifting various weights, starting from 20 kg and increasing incrementally. After each successful attempt, the weight was increased by 10 kg. Peak power output was recorded from 20 kg until the one-repetition maximum (1RM) was determined (Pacholek and Zemková, 2020). If a student failed to lift the weight, they were given one more attempt with 5 kg less weight to ensure a more accurate detection of their 1RM.

To assess strength parameters, a monitoring device, the FITROdyne Premium (FITRONIC, Bratislava, Slovak Republic), was utilized. It is a system with a sensor unit containing a linear encoder. This computer-based system for the assessment of strength capabilities and feedback monitoring of strength training. This device is capable of measuring vertical speed and range of motion, particularly during strength exercises. It includes a sensor connected to a barbell. Using data related to weight and acceleration, the system can calculate force, power, and position. The device offers immediate feedback after each repetition (Fitronic, 2017). The device is designed to comprehensively capture biomechanical parameters during workouts, including vertical velocity and the length of motion. Consisting of a sensor unit equipped with a precise encoder and reel. The system utilizes extensive computer software to facilitate the collection, calculation, and real-time display of fundamental biomechanical parameters relevant to the workout. (Pacholek and Zemková, 2020). The reliability of the parameters obtained using this system has been established in various exercises, including squat jumps and biceps curls (Jennings et al., 2005), chest presses on the bench and on a Swiss ball (Zemková et al., 2014), deadlift to high pull on the Smith machine and with free weights (Zemková et al., 2016), and standing cable wood chop exercises (Zemková et al., 2017). The testing took place in a consistent location, with the same timing and on the same days.

### 2.5 Statistical analysis

Statistical analysis was conducted using IBM SPSS Statistics 23, (IBM Corporation, United States). The basic descriptive parameters, including standard deviation and mean, were computed. The Shapiro-Wilk Test for normality was applied to assess the distribution of all variables. To analyze participant characteristics was employed a one-way analysis of variance (ANOVA) and the Kruskal–Wallis Test. To evaluate statistical changes between pre- and post-tests within groups, the paired samples *t*-test. For nonparametric data, was utilized the Related Samples Wilcoxon signed-rank test. To compare differences in strength parameters among groups a Two-Way Repeated Measures ANOVA with a Tukey *post hoc* test was used. For a practical interpretation of the research findings, was reported the effect size (ES). Cohen’s criteria for effect sizes categorize them as small (d = 0.2), medium (d = 0.5), and large (d = 0.8). Additionally, for nonparametric data, Pearson’s correlation was used, with values of r = 0.1 to 0.3 considered small, r = 0.3 to 0.5 as medium, and r = 0.5 to 1.0 as large (McLeod, 2020).

## 3 Results

The results from the characteristics of the participants (Table 1) indicate that all groups were homogeneous in terms of height, body mass, and body mass index. However, a significant difference was observed in their age (*p* = 0.003).

**TABLE 1:** Characteristic of participants (mean ± SD and n).

Variable	Whole group (n = 48	Real-time quantitative feedback group (n = 12)	Verbal encouragement group (n = 12)	Without stimulus group (n = 12)	Control group (n = 12)	*p*-value
Age (y)	20.52 ± 2.84	22.83 ± 4.20	20.67 ± 2.06	18.83 ± 0.94	19.75 ± 1.48	0.003
Height (cm)	174.63 ± 6.70	175.42 ± 6.67	175.08 ± 6.65	176.08 ± 5.65	171.92 ± 7.73	0.442
Body mass (kg)	80.08 ± 19.96	82.23 ± 18.96	81.24 ± 18.94	86.67 ± 23.67	70.18 ± 16.22	0.220
BMI (kg.m^-2^)	26.16 ± 5.96	26.58 ± 5.18	26.52 ± 6.14	27.90 ± 7.43	23.64 ± 4.64	0.362

**FIGURE 2:**
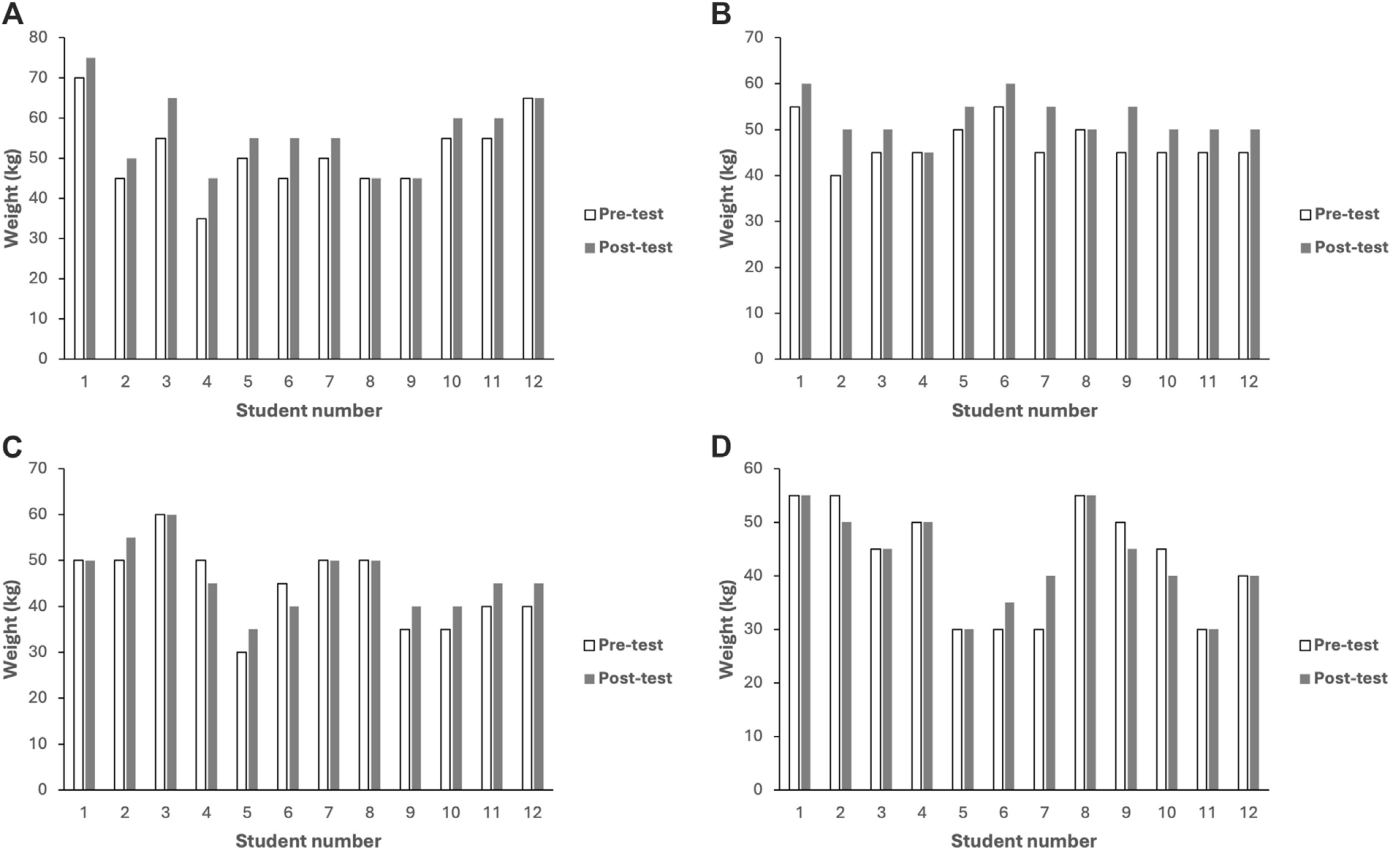
**(A)** The individual changes in one repetition maximum within the real-time quantitative feedback group. **(B)** The individual changes in one repetition maximum within the verbal encouragement group. **(C)** The individual changes in one repetition maximum within the without any external stimulus group. **(D)** The individual changes in one repetition maximum within the control group.

The changes in mean weight for one-repetition maximum (Table 2) demonstrate significant improvements in the real-time quantitative feedback and verbal encouragement groups. Following group comparisons, significant differences were identified between real-time quantitative feedback and control group groups (*p* = 0.009). Figures 2A–D illustrate the individual changes (pre-post measurement) in one-repetition maximum within each group.

**TABLE 2:** One repetition maximum in bench press exercise.

Variable	Pre-test	Post-test	Percentage of change (%)	*p*-value	d Value
Real-time quantitative feedback group	51.25 ± 9.56	56.25 ± 9.32	9.76	0.001	0.529
Verbal encouragement group	47.08 ± 4.50	52.50 ± 4.52	11.51	0.001	1.201
Without stimulus group	44.58 ± 8.94	46.25 ± 7.45	3.75	0.166	
Control group	42.92 ± 11.02	42.92 ± 9.02	0	1.000	

**FIGURE 3:**
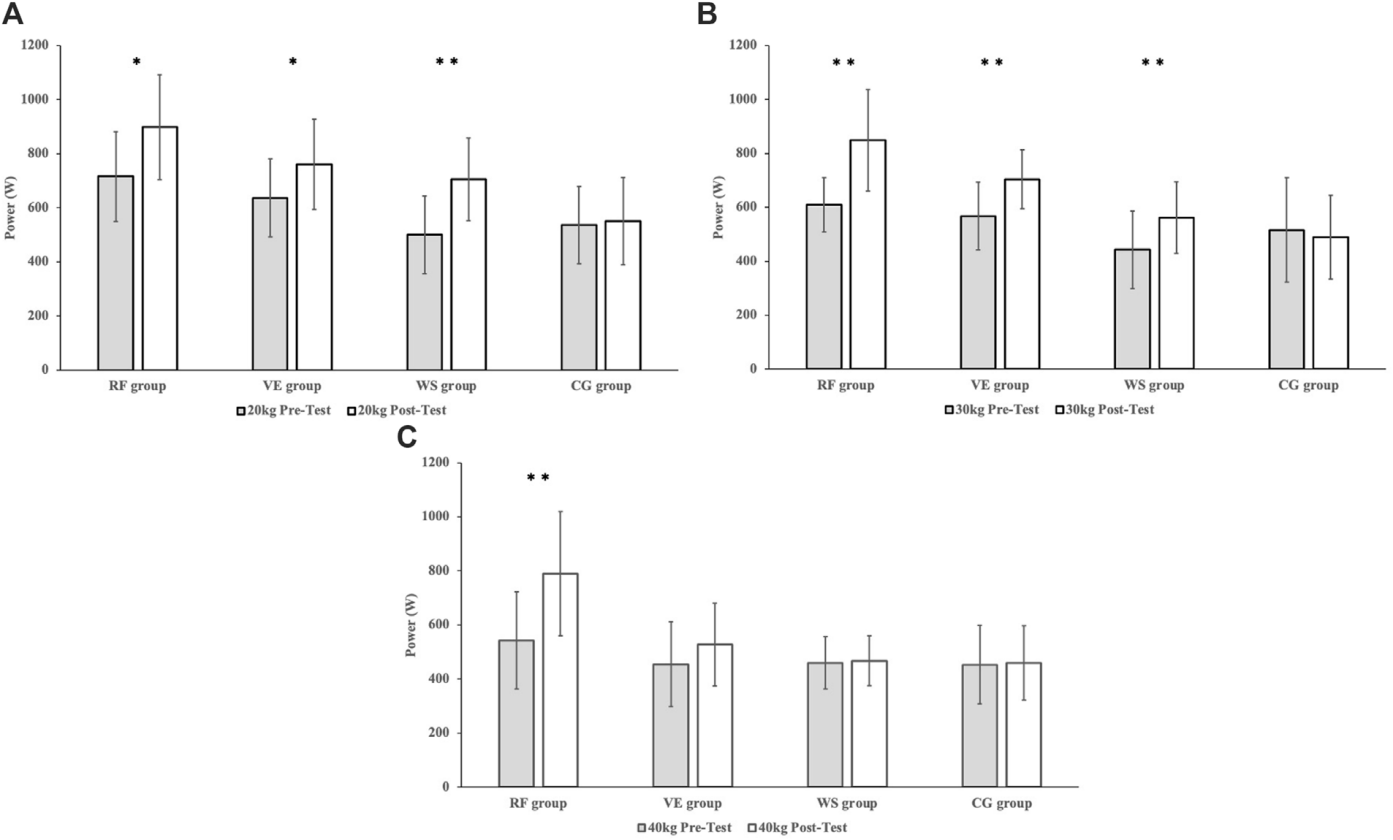
**(A)** The mean power produced during the bench press at weights 20 kg prior to and after 8 weeks of the training program (*p ≤ 0.05, **p ≤ 0.01). **(B)** The mean power produced during the bench press at weights 30 kg prior to and after 8 weeks of the training program (**p ≤ 0.01). **(C)** The mean power produced during the bench press at weights 40 kg prior to and after 8 weeks of the training program (**p ≤ 0.01).

The mean power produced during the bench press (BP) in the concentric phase at weights 20 kg (Figure 3A) significantly improved all groups except the control group. Real-time quantitative feedback group by about 183W (20,32%, *p* = 0.012, r = 0.340), verbal encouragement group by about 125W (16.43%, *p* = 0.048, d = 0.802) and without any external stimulus group by about 205W (29.11%, *p* = 0.001, d = 1.3834). Following group comparisons, significant differences were identified between real-time quantitative feedback and control group groups (*p* = 0.001), real-time quantitative feedback and without any external stimulus groups *p* = 0.004, verbal encouragement, and control groups *p* = 0.43.

At a weight of 30 kg (Figure 3B), similar results were observed with significant improvements in the real-time quantitative feedback group (239 W, 28.17%, *p* = 0.001, d = 1.581), the verbal encouragement group (137 W, 19.40%, d = 1.159), and the without any external stimulus group (120 W, 21.25%, *p* = 0.002, d = 0.862). The control group did not improve significantly. Following group comparisons, significant differences were identified between real-time quantitative feedback and control group groups (*p* = 0.002), real-time quantitative feedback, and without any external stimulus groups *p* = 0.002.

At a weight of 40 kg (Figure 3C), significant improvements were observed only in the real-time quantitative feedback group (247 W, 31.30%, *p* = 0.001, d = 1.199). The other groups did not exhibit significant improvements. Following group comparisons, significant differences were identified between real-time quantitative feedback and control group groups (*p* = 0.017), real-time quantitative feedback and without any external stimulus groups *p* = 0.017, and real-time quantitative feedback and verbal encouragement groups *p* = 0.029.

**FIGURE 4:**
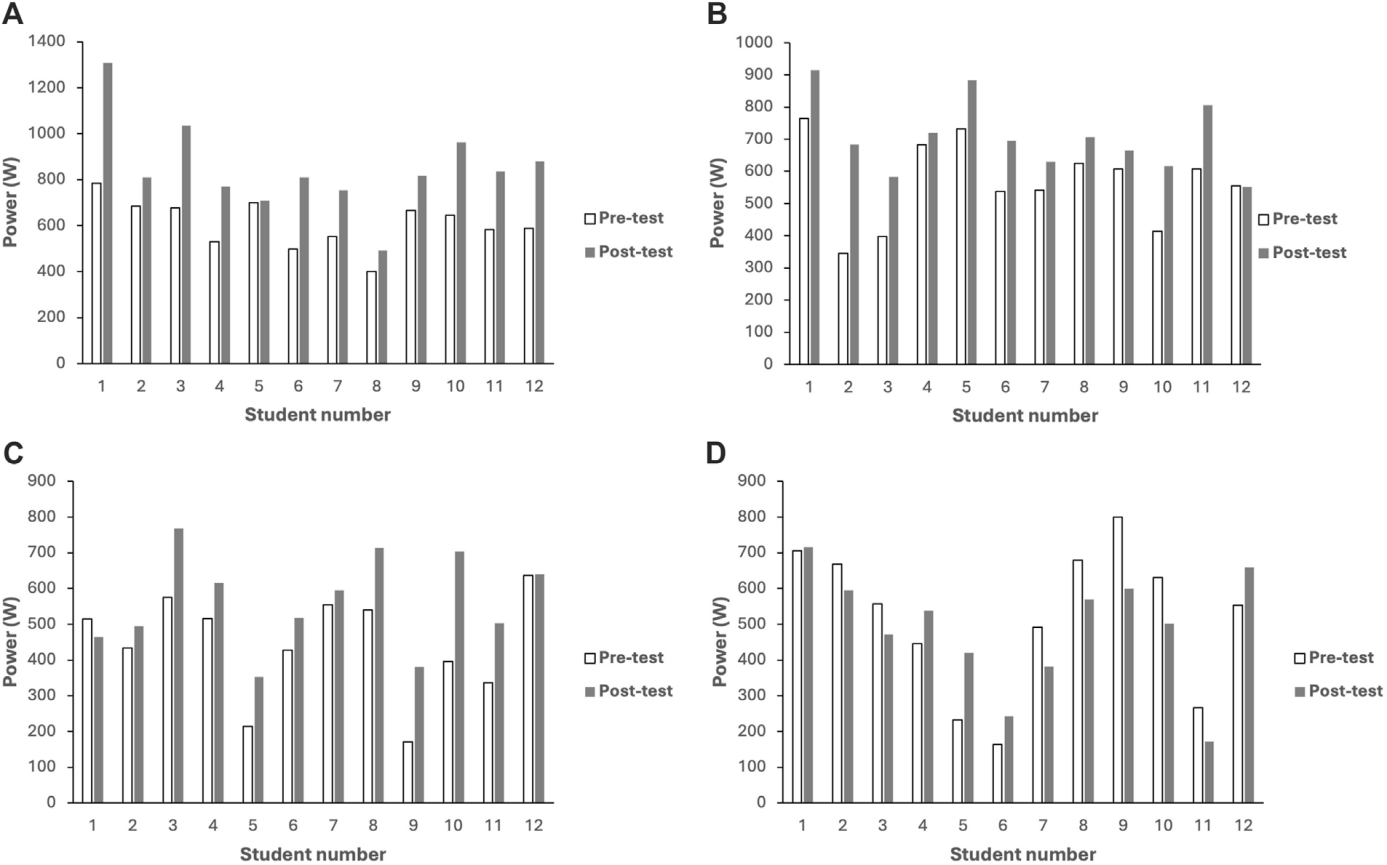
**(A)** The individual changes in maximum power (at the 30 kg weight of the barbell) within the real-time quantitative feedback group. **(B)** The individual changes in maximum power (at the 30 kg weight of the barbell) within the verbal encouragement group. **(C)** The individual changes in maximum power (at the 30 kg weight of the barbell) within the without any external stimulus group. **(D)** The individual changes in maximum power (at the 30 kg weight of the barbell) within the control group.

Following pairwise comparisons, significant differences in mean power were found between the real-time quantitative feedback and control groups (*p* = 0.004) during the bench press exercise at the weight of 20 kg. Similarly, significant differences were noted between real-time quantitative feedback and without any external stimulus groups (*p* = 0.009), real-time quantitative feedback and control groups (*p* = 0.001), verbal encouragement and control groups (*p* = 0.001), and without any external stimulus and control groups (*p* = 0.038) at the weight of 30 kg. Lastly, significant differences emerged between groups real-time quantitative feedback and verbal encouragement groups (*p* = 0.014), real-time quantitative feedback and without any external stimulus groups (*p* = 0.002), and real-time quantitative feedback and control groups (*p* = 0.003) at the weight of 40 kg. Figures 4A–D illustrate the individual changes (pre-post measurement) in maximum power within each group at the 30 kg weight of the barbell.

## 4 Discussion

The purpose of the study was to compare the effects of different stimuli on strength parameters in bench press exercises after 8 weeks of a training protocol. The main findings of this study indicate that real-time quantitative feedback and verbal encouragement groups that adhered to the protocol experienced significant improvements in maximal strength and mean power compared to the control group, thus confirming the first hypothesis. The second hypothesis was also confirmed as the real-time quantitative feedback group showed greater improvements in mean power at the weight of 40 kg compared to the other groups. The improvements in other factors, such as mean power at 20 and 30 kg and one repetition maximum, were similar to those of the verbal encouragement group. However, the without any external stimulus group only demonstrated significant improvements in mean power at 20 and 30 kg, without a corresponding improvement in maximal strength. Group comparison has shown superior results of the real-time quantitative feedback group compared to the other groups in mean power mainly at a different weight (20, 30, 40 kg). This outcome aligns with our last hypothesis that the experimental groups would exhibit significantly greater improvements in mean power than in maximal strength.

The study by Pareja-Blanco et al. (2014) documented a positive impact of maximal intended velocity compared to half-maximal concentric velocity on squat performance in a 6-week program. In this study, physically active men demonstrated a greater improvement in maximum strength (effect size: 0.94 vs. 0.54) and in velocity developed against resistance (effect size: 1.76 vs. 0.88). In this study, similar improvements in the mean power of the concentric phase were observed, particularly on lighter loads but different results were yielded particularly in heavy loads and one-repetition maximum performance on the bench press exercise. The without any external stimulus group did not exhibit a significant improvement in the mean one-repetition maximum or the mean power on heavier loads. Several factors may contribute to this discrepancy, including differences in smaller amounts of repetition (4–6 vs. 8), sets (2 vs. 3), and loads in our program. Selected exercise when the bench press utilizes relatively smaller muscle mass than the back squat (Argus et al., 2011) and statistically significantly smaller differences compared to other fitness abilities were also recorded in acute resistance training performance in the bench press exercise. The conclusion was that the performance close to maximal strength might not be sensitive enough to external stimulation (Pacholek and Zemková, 2022). Moreover, motivation could also be a crucial factor, as evidenced by the significant improvements in maximum strength parameters in the real-time quantitative feedback and verbal encouragement groups.

Weakley et al.'s meta-analysis (2023) reveals that various forms of feedback have positive effects on chronic training adaptation, particularly over a 4–6-week period. Notably, improvements were observed in key performance metrics such as velocity, power, and strength when feedback was incorporated during training sessions, in contrast to scenarios where no feedback was given. This aligns with similar findings from studies by Nagata et al. (2018), Randel et al. (2011), and Vanderka et al. (2020), which support the idea that feedback enhances performance. A noteworthy aspect of Weakley et al.'s analysis (2020) is the comparison of different feedback types when differences between verbal encouragement, kinematic feedback, and verbal kinematic feedback were generally minimal, likely to very likely trivial during the back squat exercise. This implies that, for the back squat exercise specifically, athletes may not experience significant distinctions in performance when employing these feedback methods. In contrast, the meta-analysis emphasizes the superiority of visual feedback when it comes to chronic performance gains. It was found that visual feedback had a statistically greater effect on immediate performance improvement compared to verbal feedback (Weakley et al., 2023) and from Keller et al. (2014) study is known that visual feedback decreases mean concentric barbell velocity loss, and improves perceived workload, competitiveness, and motivation. This finding aligns with the present study when real-time quantitative feedback outperformed both verbal encouragement and training without any stimuli in most of the parameters evaluated in the BP.

Pérez-Castilla et al. (2020) describe in their study that to enhance power and velocity performance during the concentric phase of the movement, it is more effective to provide feedback after each repetition rather than after each set. Furthermore, Jiménez-Alonso et al. (2022) found that this feedback is particularly advantageous when emphasizing strength over power, as it leads participants to shift their motivation from internal to external sources of information, fostering a more competitive mindset. Additionally, Weakley et al. (2020) stated that for athletes with low levels of conscientiousness, offering verbally encouraging statements after each repetition may yield the greatest benefit. In our study, we implemented real-time quantitative feedback after each repetition and provided verbal encouragement before every repetition. Furthermore, Nagata et al. (2018) achieved superior results in loaded jump abilities when providing verbal feedback about the bar’s velocity after each repetition, compared to kinetic-visual or average feedback, which was given after each set. However, the optimal approach for combining these stimuli remains a subject of inquiry. Notably, the provision of objective information about students’ performance in the real-time quantitative feedback group, compared to the verbal encouragement group, resulted in greater improvements, particularly in power parameters, regardless of whether heavy or lighter loads were used.

The study by Lee et al. (2021) indicates that verbal encouragement is effective in maintaining central activation; however, it may not be sufficient to produce significant increases in strength parameters in acute conditions. A similar study conducted by Pacholek (2021) also reported statistically non-significant results in strength parameters, particularly in the bench press exercise. In contrast, Miller et al. (2021) found that a combination of real-time feedback and verbal encouragement leads to significantly better results compared to a group that only received verbal encouragement from the investigator. On the other hand, a study by McNair et al. (1996) suggests that participants in their research significantly improved in strength parameters, although electromyograph activity remained unchanged. The effectiveness of verbal encouragement might be attributed to factors such as the presence of a teacher, their interest in the subject’s performance, and the subject’s maximal effort, as suggested by Andreacci et al. (2002). Consequently, based on these findings, the impact of verbal encouragement on acute resistance training performance remains unclear. Nevertheless, this study highlights the positive effect of verbal encouragement on chronic adaptation, particularly in the context of power and maximal strength.

The efficacy of real-time quantitative feedback as a modulating factor is underscored by its provision of objective feedback to students during their workout sessions. This feedback not only serves as a source of motivation for improved performance but also fosters a sense of competition as students compare their progress with their peers. The immediacy of information in response to any lapse in concentration plays a pivotal role in enhancing effort and execution. In contrast, verbal encouragement or without any external stimulus lacks these distinct advantages, resulting in a comparatively diminished impact compared to the dynamic and immediate influence of real-time quantitative feedback.

From a physiological point of view, we can distinguish physiological aspects based on motivation and emotion (Bradley and Lang, 2000), which could play a significant role in this research. Hormones and energy levels guide motivation; on the other hand, emotions arise from innate biological response patterns in our brains and bodies when hormones and neurotransmitters chemically affect the activity of the brain (limbic system) and its role in emotional processing (Gross and Canteras, 2012). In our scenario, it was a key point to prepare the body with a motivational stimulus to achieve better performance and focus on the task. This could happen by releasing adrenaline, which increases heart rate, blood pressure, and other physical responses (Stanton and Schultheiss, 2009). Other hormones, like dopamine and serotonin, could also play important roles in selected stimuli, as students might feel they would like to repeat this training or feel satisfaction with their accomplishments and results (Baixauli Gallego, 2017). It appears that real-time quantitative feedback influences these processes the most compared to other selected stimuli.

The study has several notable limitations. Firstly, the limited number of participants who took part in the training program raises concerns about the generalizability of the findings. Additionally, the significant age difference among the selected groups may have introduced confounding variables that could impact the results, potentially skewing the outcomes This is compounded by the significantly superior results of the real-time quantitative feedback group compared to the groups without stimuli and the control group in power at 20 kg. Furthermore, the varying number of training units completed by some participants (14th and 16th) introduces an additional variable that could have influenced the results and made it challenging to attribute improvements solely to the training protocol.

In terms of future research, it would be valuable to conduct studies with larger and more diverse participant samples and exercises to enhance the generalizability of the findings. Additionally, exploring the effects of different stimuli on muscular strength and power, particularly when tailored to specific strength parameters or fitness abilities, could provide valuable insights. Moreover, investigating whether the same stimuli have a similar effect on female students, athletes, and different age groups, including both older and younger populations, would be an interesting avenue for research. This approach could help discern potential differences in response to training stimuli among various demographic groups, contributing to a more comprehensive understanding of maximal strength and power improvements.

## 5 Conclusion

The purpose of this research was to investigate methods for enhancing motivation and performance among young, healthy participants during physical education (PE) classes at the university level. The study revealed that different stimuli have varying positive effects on students’ performance during resistance training, offering valuable insights into how to effectively encourage and motivate students to achieve better results in strength training. Real-time quantitative feedback emerged as a promising choice for PE classes, not only for its role in improving results but also for providing objective information about students’ efforts and performance. Verbal encouragement from teachers was shown to lead to greater improvements in maximal strength parameters compared to situations without any stimuli. This underscores the potential for educators to positively influence their students when objective feedback may not be readily available during physical education activities.

## Data Availability

The raw data supporting the conclusions of this article will be made available by the authors, without undue reservation.
